# Can sign language make you better at hand processing?

**DOI:** 10.1371/journal.pone.0194771

**Published:** 2018-03-28

**Authors:** Francesca Peressotti, Michele Scaltritti, Michele Miozzo

**Affiliations:** 1 Dipartimento di Psicologia dello Sviluppo e della Socializzazione, Università di Padova, Padova, Italy; 2 Dipartimento di Psicologia e Scienze Cognitive, Università di Trento, Rovereto, Italy; 3 Department of Psychology, The New School for Social Research, NewYork, NewYork, United States of America; Northeastern University, UNITED STATES

## Abstract

The languages developed by deaf communities are unique for using visual signs produced by the hand. In the present study, we explored the cognitive effects of employing the hand as articulator. We focused on the arbitrariness of the form-meaning relationship—a fundamental feature of natural languages—and asked whether sign languages change the processing of arbitrary non-linguistic stimulus-response (S-R) associations involving the hand. This was tested using the Simon effect, which specifically requires such type of associations. Differences between signers and speakers (non-signers) only appeared in the Simon task when hand stimuli were shown. Response-time analyses revealed that the distinctiveness of signers’ responses derived from an increased ability to process memory traces of arbitrary S-R pairs related to the hand. These results shed light on the interplay between language and cognition as well as on the effects of sign language acquisition.

## Introduction

The discovery that in language the relationship between meaning and form **can be** arbitrary has been of paramount importance in language research and marked the dawn of modern linguistics [1]. Arbitrariness is universal in natural languages, spoken and sign languages alike [2]. It stems primarily from the possibility of combining basic constituents in seemingly infinite arrangements, a process involving phonemes in spoken languages, and the parameters of handshape, movement, and location in sign languages [3]. Even though arbitrariness seems weakened in iconic linguistic forms that transparently express certain aspects of their meaning, it nevertheless represents a pervasive feature of natural languages. While in spoken languages the effects of arbitrariness have a long evolutionary history, in sign languages its effects have occurred within a reduced time scale and involve a different articulator: the hand. In the present study, we investigated the possible effects of associating the hand in the flexible and variable way required by the arbitrariness of sign language.

Research on the cognitive effects of sign language acquisition has primarily concentrated on vision. Findings demonstrated an enhancement of visual mental imagery [4] and facial discrimination [5], and stronger allocation of visual attention toward the inferior visual field [6] in signers compared to speakers. Because results were replicated between deaf and hearing signers, they appear to reflect the adaptation to the visual-spatial characteristics of sign language rather than sensory deprivation. More recently, sign language was shown to affect the serial position encoding of visually presented stimuli [7]. Neuroimaging findings have further revealed the neural underpinning of cognitive changes induced by sign language, for example showing a shift toward the left-hemisphere of the motion-selective area MT-MST [8, 9]. In contrast with prior investigations, we focused here on hand processing, exploring the effects of language arbitrariness using a Simon task.

In related tasks collectively referred to as Simon tasks, participants learn arbitrary stimulus-response (S-R) pairings—for example, they are instructed to respond with the left hand to red stimuli, and with the right hand to blue stimuli. Crucially, stimuli are shown either on the left or the right side, so that it varies whether or not stimuli appear in locations spatially corresponding to the response hand. As found in countless replications of the task [10, 11], responses are faster when stimuli and responses are on the same side (corresponding trials) relative to when they are on different sides (non-corresponding trials), a spatial compatibility effect commonly referred to as the Simon effect. The Simon effect has been explained as reflecting the interference on response choice induced by the conflicting spatial information associated with the stimuli [11, 12]. However, studies that examined the relationship between responses in temporally adjacent trials revealed that mechanisms functionally equivalent to priming could also contribute to the Simon effect [13, 14]. Responses can be especially fast when a corresponding trial immediately follows an identical corresponding trial. While these results underscore the importance of analyses focusing on first-order trial sequences, they also indicate that performance in this task in part depends on forming memory traces that consolidate S-R pairs and that facilitate the response if retrieved in the next trial. We specifically focused on the formation of these memory traces, examining whether the acquisition of sign language would affect the strength of memory traces related to arbitrary associations involving the hand.

**Fig 1:**
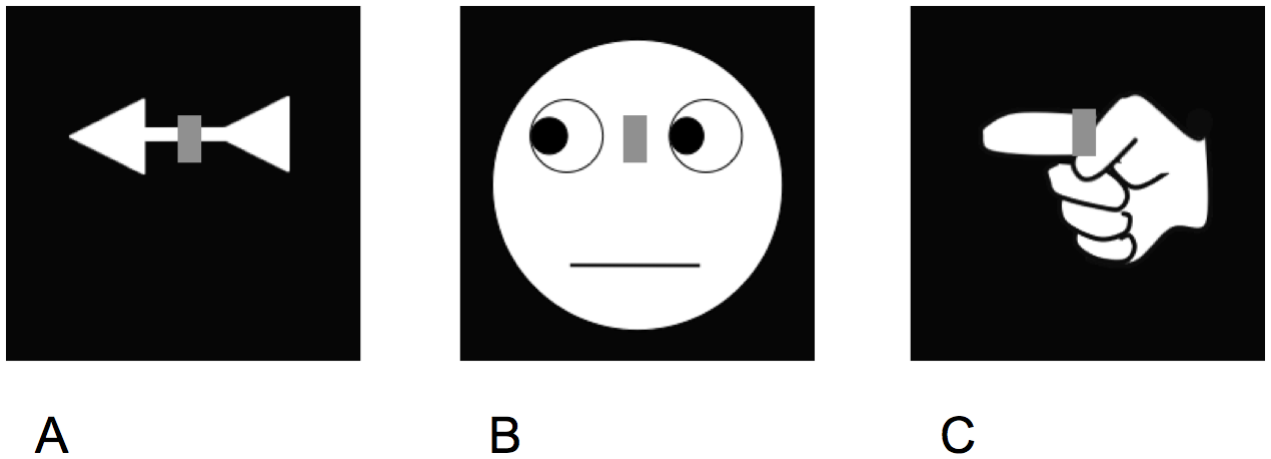
Examples of stimuli presented to induce the Simon effect. The examples shown here cue a left location (other stimuli cued a right location). Locations were cued by the arrow (A), the eye gaze (B), or the pointing hand (C). The superimposed rectangle (target) was shown either in red or green and responses varied as a function of target colors. It should be noted that the left location was cued by the left hand; direction and effector were always spatially compatible.

The Simon effect has been replicated with stimuli explicitly indicating left or right directions, including arrows [15], eye gaze [16, 17], and pointing hands [18]. In these task variants, colored stimuli appeared on the center, and participants responded with the left or the right hand depending on the stimulus color. The arrows, eyes, and hands were shown in the background and served as distractors to induce the spatial compatibility effect (see Fig 1). We tested these task variants with signers and speakers unfamiliar with sign languages. Possible effects of sign language can be of two types. Differences between signers and speakers could appear in each of these task variants. If the effects of sign language result in a generalized facilitation of hand motor processing, in line with previous findings [13, 14] we anticipate a strengthening of the memory traces associating the arbitrary S-R pairs in each of the task variants. Alternatively, differences between signers and speakers could be limited to the condition in which the hand is presented as distractor. If the effects of sign language are primarily limited to improving the processing of its articulator, the memory traces associating the arbitrary S-R pairs should be especially strong only with the hand distractor. This finding would suggest a more specific effect of sign language, one linked to hand visual processing that characterizes comprehension in sign language. The analyses we presented below aimed to specifically test these possible outcomes and focused on examining the Simon effect and the first-order trial sequences that modulate it, as shown by prior investigations [13, 14].

### Methods

#### Ethical statement

The procedures have been approved by the Ethical Committee for Psychological Research of Padua University. Written informed consent was obtained from participants or from parents in the case of minors.

#### Participants

One group of participants included 32 signers (14 female), of ages ranging from 14 to 22 years (mean = 18.0). All of them were deaf, either since birth (23) or before the age of 2 years (8) or 6 years (1), and used Italian Sign Language as their primary means of communication. A second group of participants included 32 Italian speakers (26 female), with no knowledge of any sign language (age range = 20–27 years, mean = 22.9 years).

#### Materials, procedure, and analyses

Stimuli were modeled after Dalmaso et al. (2013) [19]. A colored rectangle (the target) was presented at fixation and superimposed on a schematic white picture showing an arrow, a face, or a hand (Fig 1). The arrow and the hand pointed either to the right or the left, and the eyes in the face turned to one side or the other. Schematic illustrations of both the right and the left hand were shown. The right and left hand pointed to the right and left direction, respectively, so that effector and direction were spatially compatible. The pointing hand shown as distractor is not a sign in the Italian sign language used by our signing participants—it is just a pointing gesture, as for the speaking participants. With equal probabilities, targets appeared in red or green, and pictures cued the left or right locations.

On each trial, a fixation point appeared in the center of the screen for 675 ms, and was replaced by the picture and the superimposed target, which stayed on view until the response was initiated or for up to 3 s. A blank screen appeared during inter-trial (500 ms), unless the response was either incorrect or missed, in which case an error message was shown. Participants were instructed to respond to each color by pressing a letter on the keyboard with a specific hand. The letters A and L were chosen because of their left and right location on the keyboard. Each distractor (arrow, eyes, hand) was tested with 120 trials presented in a randomized order within three blocks. A self-terminated break occurred after 60 trials within each block. The testing of each distractor condition was preceded by 16 practice trials. The order in which the distractors were presented was randomly selected for each participant. The color assigned to each hand was counterbalanced across participants.

Accuracy was analyzed using generalized linear mixed-effects models, whereas correct response latencies were analyzed using linear mixed-effects models. All analyses were carried out with the lme4 package (version 1.1–14) [20] available in R [21]. All models included the fixed effects and their interactions, as well as participant random effect. Response times (RTs) faster than 200 ms were considered as anticipations and removed from analyses (*n* = 10; 0.0004% of the responses). RTs were reciprocally transformed (1000/RT) in order to reduce skewedness and better approximate normality for the distribution of models’ residuals [22]. Fixed effects were examined using chi-square deviance tests in which a model that included all the fixed effects and their interactions was contrasted with alternative models that excluded the fixed effect under examination (package car version 2.1_15 [23]). Follow-up, pairwise comparisons between conditions were conducted by applying false discovery rate correction for multiple comparisons via the package multcomp (version 1.4–6 [24]). The effect size is described for these comparisons, as the percentage (%) change obtained with RT reciprocals.

### Results

#### Simon effect

We first determined if the Simon effect varied across groups (signers vs. speakers) and distractor (arrow vs. eyes vs. hand). To this end, we used a model that included the fixed effects of Group, Distractor, and Correspondence (spatially corresponding vs. non-corresponding S-R pairs), as well as their interactions.

Errors accounted for 3% of the responses. Error analyses revealed an effect of Correspondence (χ^2^[1] = 50.21, p < .001), reflecting the fewer errors induced by corresponding relative to non-corresponding S-R pairs (2% vs. 4%). There was also a significant Distractor by Correspondence interaction (χ^2^[2] = 12.74, p = .002)), due to a greater error occurrence, in non-corresponding S-R pairs, with the arrow distractor (6%; *b* = -1.25, *SE* = 0.18, z = -7.09, p < .001) relative to the eyes distractor (3%; *b* = 0.72, *SE* = 0.26, z = 2.80, p = .005) and the hand distractor (4%; *b* = 0.81, *SE* = 0.24, z = 3.31, p < .001).

**Fig 2:**
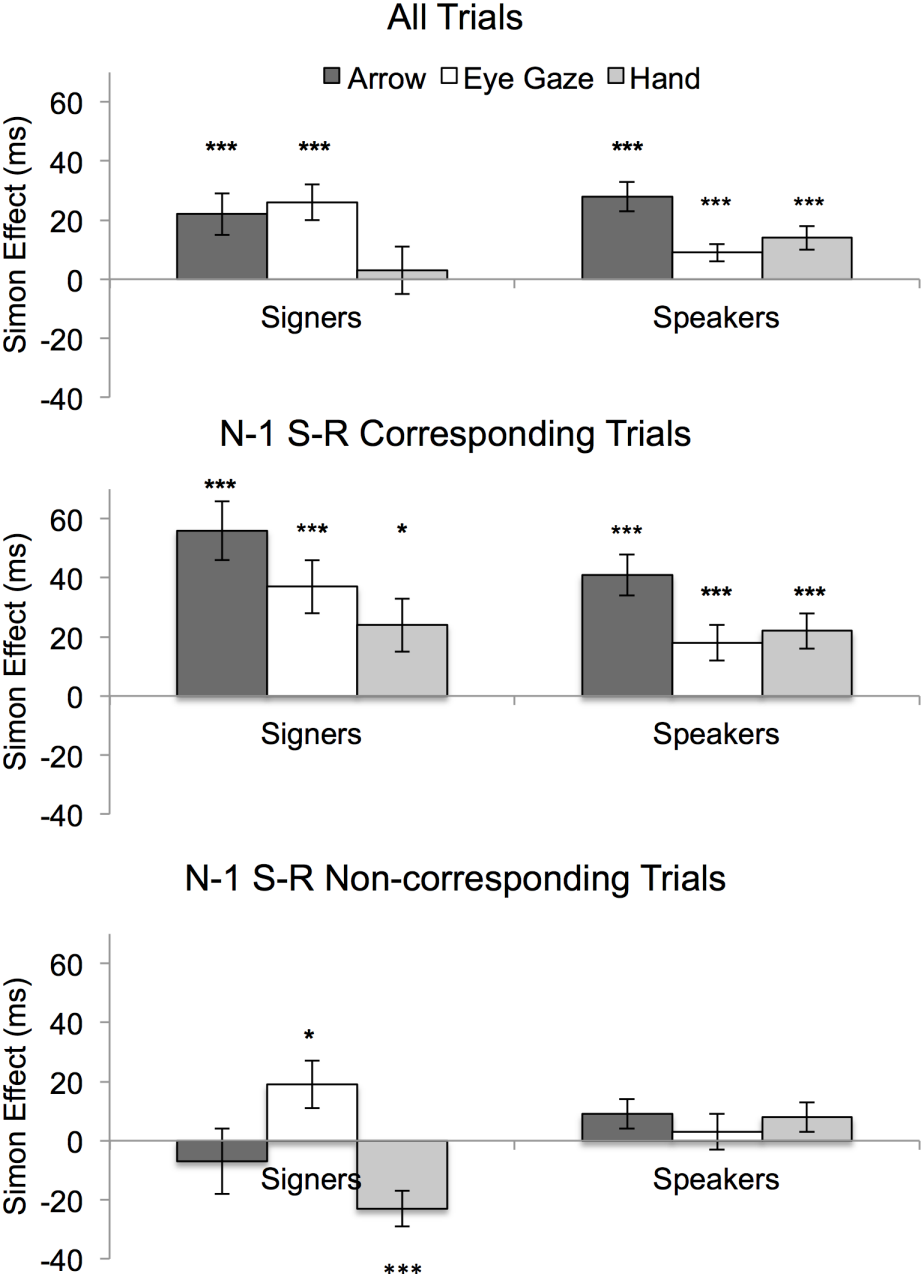
Simon effects (faster RTs to spatially corresponding S-R pairs) found across groups (signers and speakers) and distractors (arrow, eyes, hand). Analyses were based on all trials (top), or only trials immediately preceded by corresponding S-R pairs (middle) or non-corresponding S-R pairs (bottom). The lack of the Simon effect demonstrated by signers in the hand condition (top panel) reflected the opposing effects of spatial congruency induced by N-1 trials. * p < .05, *** p < .001.

A summary of correct RTs is presented in Table 1 and Fig 2. RTs were significantly slower for signers (χ^2^[1] = 21.93, p < .001), a result that aligns with a number of prior findings that showed slower RTs with deaf participants in a wide range of tasks (e.g., [25]). The 3-way interaction (χ^2^[2] = 7.57, p = .02) indicated group differences in the Simon effect. With speakers, the Simon effect demonstrated by slower RTs for non-corresponding S-R pairs appeared with the distractors arrow (*b* = 0.11, *SE* = 0.02, z = 7.29, p < .001; % change = 5.91), eyes (*b* = 0.04, *SE* = 0.02, z = 2.77, p = .007; % change = 2.11), and hand (*b* = 0.06, *SE* = 0.02, z = 4.01, p < .001; % change = 3.08). With signers, the Simon effect was found with the distractors arrow (*b* = 0.12, *SE* = 0.02, z = 7.74, p < .001; % change = 5.53) and the eyes (*b* = 0.08, *SE* = 0.02, z = 5.34, p < .001; % change = 3.81) but not the distractor hand (*b* = 0.02, *SE* = 0.02, z = 1.13, p = .26; % change = 0.78). The difference between the Simon effect generated by the hand distractor in Speakers compared to Signers closely approached conventional significance (*b* = 0.05, *SE* = 0.02, z = 2.02, p = .051; % change = 71.52).

**Table 1:** 

Distractor	Signers S-R Correspondence			Speakers S-R Correspondence		
	Yes	No	Difference	Yes	No	Difference
Arrow	500	522	22	417	445	28
Eyes	480	506	26	442	451	9
Hand	510	512	2	440	454	14

Mean RTs for correct responses for each group of participants according to conditions

In summary, RTs revealed group differences only with the hand distractor, reflecting the lack of the Simon effect with signers.

#### Sequential analyses

The Simon effect could be absent with signers because the pointing hand did not represent an effective spatial distractor. This could derive from indirect activation of additional linguistic information induced by the hand distractor. Alternatively, the reduced cuing effect of the hand could be related to the multiple spatial systems exploited in sign language—signer-centered for production, viewer-centered for comprehension—in which opposite directions are encoded for the pointing hand.

Sequential analyses were carried out to evaluate the hypothesis of a reduced cuing effect. Prior studies showed that the Simon effect varied as a function of the immediately preceding (N-1) trial. The Simon effect, robust after corresponding trials, was reduced or absent after non-corresponding trials [14, 26]. Were the cueing effect reduced with the hand distractor, these findings would not appear with signers. The Simon effect induced by the hand distractor would in fact be weak or absent in *both* types of trials (corresponding and non-corresponding). The hypothesis of a reduced cuing effect does not anticipate finding the Simon effect in the corresponding trials only. That is, a replication of prior findings in the hand condition with signers would be a type of evidence inconsistent with such hypothesis. We expected nevertheless to replicate prior findings in the other task conditions.

For sequential analyses, we removed the first response of each block and the first response after breaks. Further, because of possible post-error slowing, responses that followed an error were discarded as well. Responses from signers and speakers were analyzed separately using the fixed effects N-1 (corresponding vs. non-corresponding), Correspondence, and Distractor, in addition to the by-participants random intercepts. With signers, the Simon effect varied as a function of N-1 trials (N-1 x Correspondence: χ^2^[1] = 35.80, p < .001), although, as shown in Fig 2, it occurred differently across distractors (N-1 x Correspondence x Distractor: χ^2^[2] = 11.52, p = .003). With the hand distractor, the Simon effect appeared after N-1 corresponding trials (*b* = 0.10, *SE* = 0.02, z = 4.43, p < .001; % change = 4.25) but reversed after N-1 non-corresponding trials (*b* = -0.08, *SE* = 0.02, z = -3.33, p = .001; % change = -3.19). By contrast, with arrow and eyes distractors the Simon effect was present after N-1 corresponding trials (ps < .001) but absent or reduced after N-1 non-corresponding trials. Prior findings were thus replicated with signers with arrow and eyes distractors. With speakers, N-1 trials also modulated the Simon effect (N-1 x Correspondence: χ^2^[1] = 31.62, p < .001): for all distractor types the Simon effect appeared in N-1 corresponding trials (ps < .001) but was lacking in N-1 non-corresponding trials (ps > .12). The results from speakers therefore aligned with those from previous studies. There was also evidence that the Simon effect varied across distractors in N-1 corresponding trials (N-1 x Correspondence x Distractor: χ^2^[2] = 7.33, p = .03), a result that, as shown in Fig 2, was due to the greater effect induced by the arrow distractor relative to the eyes distractor (b = 0.11, SE = .03, z = 3.95, p < .001) and the hand distractor (b = 0.09, SE = .03, z = 3.26, p = .002). This latter result possibly reflects the greater strength of the arrow as a direction cue, a sign specifically designed and universally used to indicate direction.

In summary, sequential analyses shed light on the lack of the Simon effect signers demonstrated in the hand condition. First, the replication of prior findings with the hand distractor in N-1 corresponding trials demonstrated that the pointing hand was an effective spatial distractor. Second, the Simon effect was absent because of the opposing effects of N-1 trials that cancelled out each other.

#### Repeated trials

**Fig 3:**
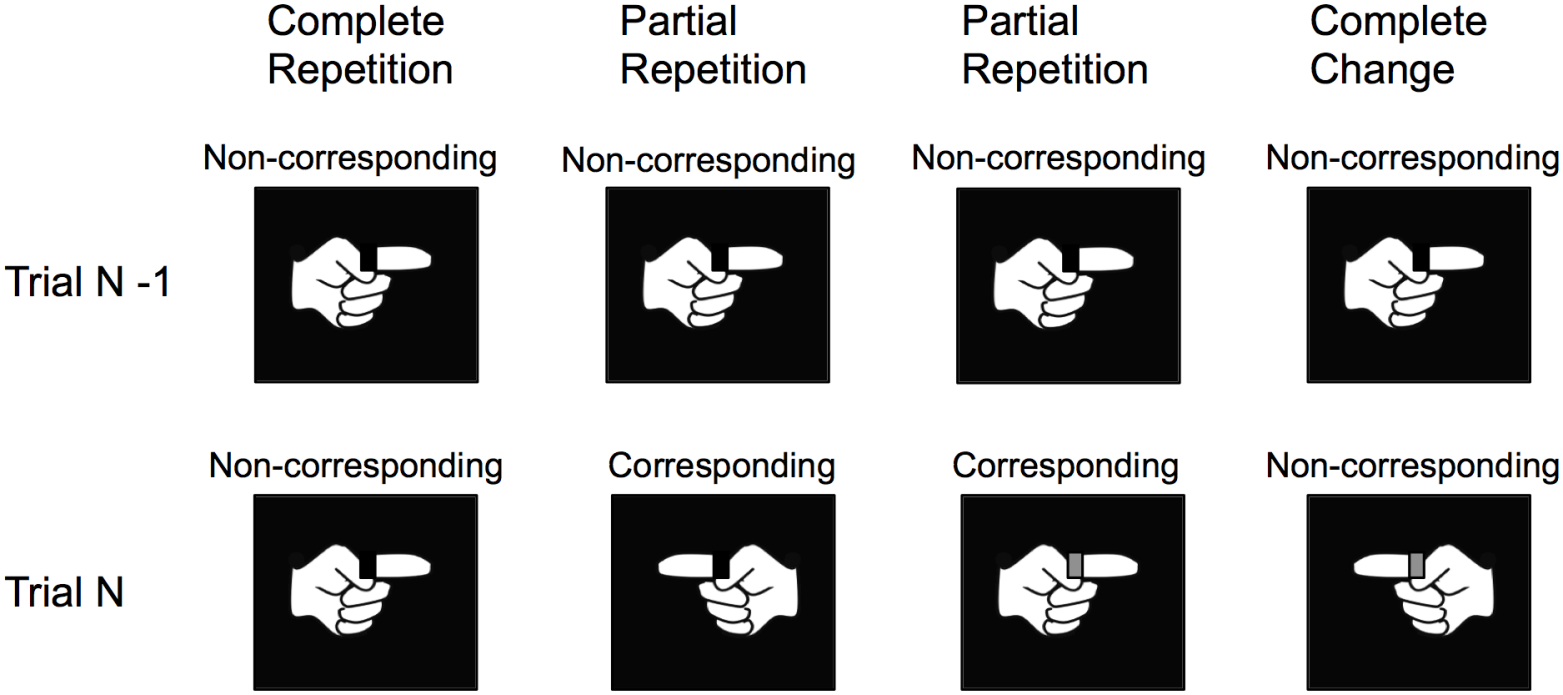
The relations between stimuli and responses occurring in adjecent trials is illustrated here assuming left-hand responses to dark targets and right-hand responses to light targets. In the example shown here, stimuli and responses were always non-corresponding in the preceding trial (N-1). Stimuli and responses in the next trials (N) were either repeated—completely or in part—or changed entirely.

As shown in Fig 3, the relation between stimuli and responses occurring in consecutive trials varied, since stimuli and responses were either repeated—completely or in part—or changed entirely.

**Fig 4:**
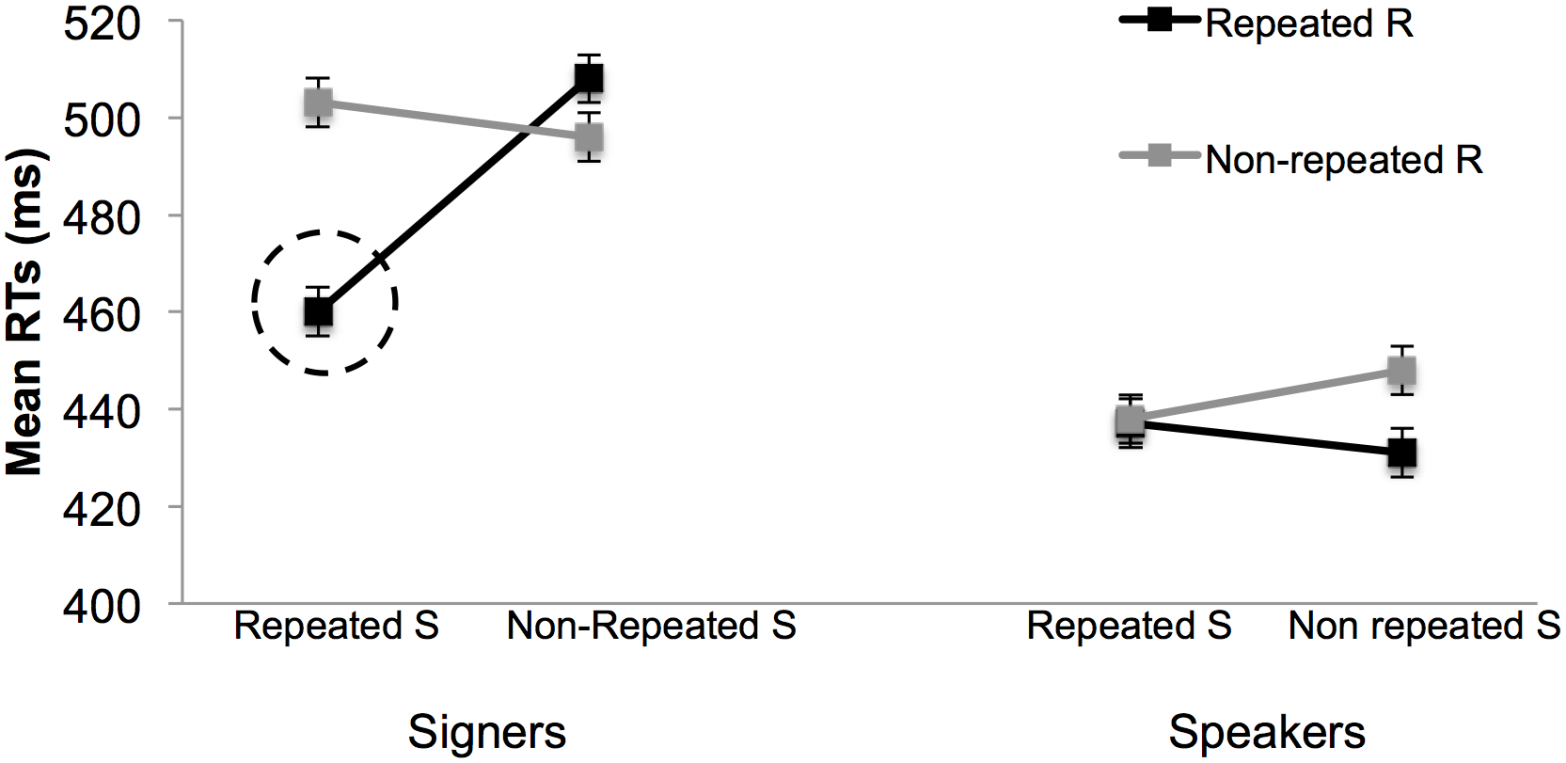
Effects of stimulus (S) and response (R) repetition in N-1 non-corresponding trials (hand condition). Signers were especially fast when stimuli and responses were jointly repeated between consecutive trials.

We examined whether this variation was related to the reversal of the Simon effect signers demonstrated with the hand distractors in N-1 non-corresponding trials. We therefore analyzed the variables Stimulus (repeated vs. non-repeated) and Response (repeated vs. non-repeated). The Stimulus x Response interaction (χ^2^[1] = 17.29, p < .001) reflected signers’ fast responses to repeated N-1 non-corresponding trials that displayed the hand distractor. This repetition effect was unique to signers, as shown in Fig 4 and confirmed by the Stimulus x Response x Group interaction (χ^2^[1] = 16.49, p < .001) found with the hand distractor in N-1 non-corresponding trials. Furthermore, it was only with the hand distractor that responses from signers and speakers differed after N-1 non-corresponding trials, as indicated by the lack of Stimulus x Response x Group interaction (ps > .19) with the distractors arrow or eyes. Moreover, group differences were absent with N-1 corresponding trials for all the distractors (ps > .22)—here, fast responses occurred with complete repetitions and complete changes, as shown by the the Stimulus x Response interactions found with across all distractors (ps < .001) reflected the obtained for both groups.

In summary, analyses of S-R repetition revealed that the lack of the Simon effect found with signers in the hand condition resulted from the fast responses to completely repeated S-R pairs in N-1 non-corresponding trials. Crucially, this type of result was observed only with signers and with the hand distractor.

## General discussion

The Simon effect we consistently found with the arrow and the eye gaze was peculiarly absent with signers in the hand condition. This anomalous result was traceable to signers’ unique sensibility to the repetition of spatially incompatible S-R pairs that made their responses to this type of stimuli especially fast. These quick responses effectively cancelled out the advantage of spatially compatible S-R pairs, thus determining the lack of the Simon effect observed with signers in the hand condition. It is worth emphasizing that signers’ responses differed only with spatially incompatible S-R pairs in the hand condition. In all the other conditions, their responses closely paralleled those obtained with speakers or reported in prior experiments [15, 16].

The fact that the Simon task requires hand responses provides a viable opportunity to investigate possible effects of sign language on hand motor processes. The use of the hand as articulator in sign language production could enhance hand action and control, raising the possibility that signers would perform better in the conflicting hand responses engendered by the Simon task. Our finding that in multiple conditions of the Simon task signers’ performance was in all respects comparable to speakers’ does not indicate a specific effect of sign language on hand action and control. Although conclusions should be drawn cautiously from single results, our findings are nevertheless suggestive that sign language has limited effects on the control of hand movements.

By contrast, the finding that signers’ performance differed specifically from speakers’ in the hand condition reveals that sign language is more likely to affect the processing of visually presented hand stimuli. In other words, the uniqueness of signers’ performance in the Simon task appears to be related to the visual nature of sign comprehension. In this respect, our findings echo prior results that showed that partially different brain areas support language comprehension in signers and speakers, reflecting the different modalities employed for comprehension in each type of language [9, 27, 28].

The advantage in the Simon task for spatially compatible S-R pairs was in part determined by the fast responses occurring when such pairs are repeated in consecutive trials. Although interpretable in different ways [13], the repetition effect observed in the Simon task implies the formation of a memory trace of an event that is retrieved in the successive trial. Only for signers, and only with the hand distractor, the repetition of spatially incompatible S-R pairs sped responses. While the repetition effect found in the hand condition implies that signers demonstrated a distinctive ability to form memory traces of spatially incompatible S-R pairs, the specificity of the effect on the hand condition further implies that the repetition effect was related to sign language acquisition. In line with what was discussed above, the memory trace uniquely formed by signers is based on visual representations of the hand that, as a result of an extended practice of sign language, can be easily created and integrated in the representation of the event. The hand representations processed by speakers do not have the same quality and, similarly to the representations created for other distractors shown in the task, led to repetition effects only with compatible S-R pairs. If we consider that the Simon task demands forming color-hand associations that are arbitrary, the differences found between signers and speakers suggest that arbitrary associations involving the hand established by signers apply to a broader range of conditions than those available to speakers. In other words, the formation of memory traces after non-corresponding events reveals the enhanced ability of signers to set up new associations between a given visual representation of the hand and a given stimulus (in this case a color).

Effects of sign language have been previously demonstrated on visual processing and visual imagery [6, 7, 8, 9], and our results contribute to this literature by showing effects extending to hand visual processing. Hypotheses about the specific language features responsible for these cognitive changes are necessarily speculative. A link to arbitrariness, a universal feature of languages, was proposed for our findings given the arbitrary nature of the S-R associations in the Simon task. It is probably unlikely that changes in cognitive processing are traceable to a single feature of language. It is perhaps not a coincidence that an influence was found in the Simon task where spatial position is so critical. Hand localization is one of the parameters characterizing the signs and is extensively exploited in sign languages for encoding aspects of meaning, grammatical constituents, metaphorical concepts, and topographical relations [2]. Another distinguishing spatial feature of sign languages is represented by referential loci (R-loci) in which the pointing hand is used to establish and maintain reference across a discourse [29]. The use of space is so pervasive in sign languages—as demonstrated by hand localization and referential loci—that it might have also contributed to signers’ distinctive performance in the Simon task. The effects of sign language observed with the hand distractor were nevertheless restricted to non-corresponding trials in which the hand distractor pointed to an opposite direction than the response side. Indeed, the responses of signers with corresponding trials were qualitatively equivalent to speakers’ for showing significant facilitation when the hand distractor and the response matched spatially. Signers’ responses with the hand distractor not only underscore the specificity of effects possibly rooted in language arbitrariness, but they also reveal that sign language is unlikely to affect the formation of associations based on iconic signs like the pointing hand that convey transparent and predictable spatial meanings. In other words, the fact that here the iconic sign shares the hand with linguistic signs does not prevent signers to take advantage of established stimulus-response associations to the same degree as speakers do.

Our findings provide yet another illustration that the articulator a language employs influences cognitive processing. The cognitive effects of the oral articulator, prominent at various points during the evolution of spoken languages, are now difficult to discern within a neurocognitive system in which language and cognition are so inextricably integrated. Fortunately, sign languages represent a feasible model for unveiling aspects of the language-cognition interplay that are otherwise lost in the long evolution of spoken languages.

## Supporting information

S1 File Raw data obtained in the present study. (XLSX)
